# Telomerase activation cooperates with inactivation of p16 in early head and neck tumorigenesis

**DOI:** 10.1054/bjoc.2000.1647

**Published:** 2001-02

**Authors:** J C Soria, L Morat, F Commo, D Dabit, S Perie, L Sabatier, P Fouret

**Affiliations:** Service d'Anatomie Pathologique (Pr. P. CALLARD), Hôpital Tenon, UFR Saint-Antoine, Paris, France; CEA, DSV/DRR/Laboratoire de Radiobiologie et Oncologie BP6, Fontenay-aux-Roses, France; Service d'ORL et de Chirurgie de la Face et du Cou (Pr. J. LACAU-ST-GUILY), Hôpital Tenon, UFR Saint-Antonie, Paris, France; Service d'Anatomie et de Cytologie Pathologiques (Pr. Y. LE CHARPENTIER), Groupe Hospitalier Pitié-Salpêtrière, UFR Pitié-Salpêtrière, Paris, France

**Keywords:** telomerase, p16, expression, squamous-cell carcinoma, head and neck

## Abstract

Alteration of the p16/pRb pathway may cooperate with telomerase activation during cellular immortalization and tumour progression. We studied p16 expression status by immunohistochemistry and telomerase activity using the TRAP assay in 21 premalignant lesions of the head and neck epithelium as well as 27 squamous-cell carcinomas. We also examined expression of other components of the pathway (cyclin D1 and pRb) as well as presence of human papillomavirus genomes which can target these molecules. 4 of 9 mild dysplastic lesions (44%), 8 of 12 moderate/severe dysplastic lesions (67%), and 25 of 27 squamous-cell carcinomas (92%) demonstrated high telomerase activity (*P* = 0.009). There was a parallel increase with severity of lesions for the trend in proportions of cases demonstrating p16 inactivation or cyclin D1 overexpression (*P* = 0.02 and *P* = 0.01, respectively). For Ki67, a marker of cell proliferation, this trend was not significant (*P* = 0.08). Human papillomavirus infection was only found in 4 cases among the 48 samples tested (8.3%). In conclusion, progression of disease is accompanied by a parallel and continuous increase in telomerase activity and alterations in cell cycle regulators (p16, cyclin D1), as proposed by in vitro models. © 2001 Cancer Research Campaign http://www.bjcancer.com


British Journal of Cancer (2001) **84**(4), 504–511
© 2001 Cancer Research Campaign
doi: 10.1054/ bjoc.2000.1647, available online at http://www.idealibrary.com on IDEAL®

### Telomerase activation cooperates with inactivation of p16 in early head and neck tumorigenesis

**JC Soria[1,2], L Morat[2], F Commo[1], D Dabit[1], S Perie[3], L Sabatier[2] and P Fouret[4]**

[1]Service d'Anatomie Pathologique (Pr. P. CALLARD), Hôpital Tenon, UFR Saint-Antoine, Paris, France; [2]CEA, DSV/DRR/Laboratoire de Radiobiologie et Oncologie BP6, Fontenay-aux-Roses, France; [3]Service d'ORL et de Chirurgie de la Face et du Cou (Pr. J. LACAU-ST-GUILY), Hôpital Tenon, UFR Saint-Antonie, Paris, France; [4]Service d'Anatomie et de Cytologie Pathologiques (Pr. Y. LE CHARPENTIER), Groupe Hospitalier Pitié-Salpêtrière, UFR Pitié-Salpêtrière, Paris, France

**Summary** Alteration of the p16/pRb pathway may cooperate with telomerase activation during cellular immortalization and tumour progression. We studied p16 expression status by immunohistochemistry and telomerase activity using the TRAP assay in 21 premalignant lesions of the head and neck epithelium as well as 27 squamous-cell carcinomas. We also examined expression of other components of the pathway (cyclin D1 and pRb) as well as presence of human papillomavirus genomes which can target these molecules. 4 of 9 mild dysplastic lesions (44%), 8 of 12 moderate/severe dysplastic lesions (67%), and 25 of 27 squamous-cell carcinomas (92%) demonstrated high telomerase activity ($P = 0.009$). There was a parallel increase with severity of lesions for the trend in proportions of cases demonstrating p16 inactivation or cyclin D1 overexpression ($P = 0.02$ and $P = 0.01$, respectively). For Ki67, a marker of cell proliferation, this trend was not significant ($P = 0.08$). Human papillomavirus infection was only found in 4 cases among the 48 samples tested (8.3%). In conclusion, progression of disease is accompanied by a parallel and continuous increase in telomerase activity and alterations in cell cycle regulators (p16, cyclin D1), as proposed by in vitro models. © 2001 Cancer Research Campaign http://www.bjcancer.com

**Keywords**: telomerase; p16; expression; squamous-cell carcinoma; head and neck

During malignant transformation, cancer cells acquire genetic changes through a multistep process. Maintenance of telomeres, the very ends of chromosomes, is predicted to play the key role for bypass of senescence – a process which can be prematurely induced or overcome by a number of oncogenes (Serrano et al, 1997; Lin et al, 1998; Zhu et al, 1998). High levels of telomerase activity are observed in almost all cell lines and 80–90% of human tumours (Shay and Bacchetti, 1997). In contrast, only low levels of telomerase activity are detected in normal tissues, mainly within lymphocytes and physiologically regenerating epithelial cells (Kolquist et al, 1998). The correlation between telomerase activation during cellular immortalization and activation in tumours has led to the view that telomerase activation is required for tumour growth.

P16 is specified by the α transcript containing exons 1α, 2 and 3 of the INKa/ARF locus (Quelle et al, 1995). P16 inhibits the association between CDK4/6 and D-type cyclins, and thereby blocks cyclin-D-directed CDK4/6 phosphorylation of pRb, prevention exit from $G_1$ phase (Planas-Silva and Weinberg, 1997). The role of p16 in tumorigenesis is well established (Serrano et al, 1996). P16 knockout mice are tumour prone. P16 inactivation is seen in a broad spectrum of human cancers, particularly those resulting from exposure to carcinogens, including up to 80% squamous-cell carcinomas of the head and neck epithelium (SCCHN) (Reed et al, 1996; Jares et al, 1997; Olshan et al, 1997; Papadimitrakopoulou et al, 1997; Pande et al, 1998). Alternatively, deregulation of the $G_1$/S checkpoint is achieved through pRb deletion, cyclin D1 overexpression or CDK4 mutation (Yoo et al, 1994; Barthova et al, 1995; Okami et al, 1999). Loss of p16 function may be involved in escape from the normal limits on cellular proliferative life span (Huschtscha and Reddel, 1999). There is evidence for the specific inactivation of p16 upon human keratinocyte immortalization (Munro et al, 1999).

It has been shown in vitro that both pRb/p16INK4a inactivation and telomerase activity are required to immortalize epithelial cells (Kiyono et al, 1998). Human squamous-cell carcinoma (SCC) keratinocytes immortalization requires the targeting of 4 pathways including inactivation of p16 and another involving a repressor of telomerase activity (Loughran et al, 1997). 14-3-3sigma-dependent bypass of senescence in human primary keratinocytes is accompanied by maintenance of telomerase activity and by down-regulation of the p16 tumour suppressor gene (Dellambra et al, 2000). Significant reductions in tumour formation in vivo and oncogenic potential in vitro are observed in late generations of mice doubly null for the telomerase RNA(mTR) and the INK4a tumour suppressor gene (Greenberg et al, 1999). However it is not clear whether telomerase activation is linked to p16 inactivation in human tumours.

The goal of our study was to evaluate whether progression of disease is accompanied by parallel changes in telomerase activity and alterations of components of the pRb pathway. The underlying hypothesis was that telomerase activation and alterations of the cell cycle regulators cooperate during tumour progression. Therefore, we studied telomerase activity levels as determined by the semi-quantitative TRAP assay in parallel to p16 expression in premalignant and malignant cells as determined by immunohistochemistry. Since p16 is only one player of the p16/pRb pathway, we have also evaluated by immunohistochemistry other

*Received 28 July 2000*
*Revised 2 November 2000*
*Accepted 8 November 2000*

*Correspondence to:* P Fouret components of this pathway (i.e. pRb and cyclin D1). Moreover and since the p16/pRb pathway can also be targeted by the E7 gene product of oncogenic human papillomaviruses (HPV) (Jones and Munger, 1996), we have also performed a PCR-detection of HPV genome in our samples.

### PATIENTS AND METHODS

### Patients

The premalignant and malignant specimens of the head and neck epithelium were archived tissue samples of surgically biopsied lesions from 46 patients treated at Hospital Tenon, Paris, France between 1996 and 1998. After biopsy, about half of each specimen had been embedded in OCT (Tissue-Tek: Miles, Elkhart, IN) and quick frozen in liquid nitrogen. The remaining half had been fixed in 10% buffered formalin for 24 hours or less and routinely embedded in paraffin. Sections were made 2–3 weeks prior to performing the immunohistochemistry.

Cases were selected on the basis of histological criteria. Premalignant lesions were recognized on the basis of described criteria (Crissman and Zarbo, 1989) and classified by mutual agreement between two observers (PF and J-CS) in the following groups: mild, moderate and severe dysplasia. Cases for which the histological presentation differed between paraffin-embedded and frozen sections were excluded. All premalignant paired paraffin-embedded and frozen specimens during the two-year period were consecutively included. The 21 premalignant lesions were from 20 cancer-free patients. One specimen of mild dysplasia was obtained from case 8 for whom a previous moderate dysplasia specimen was also available (metachronous presentation). The 27 SCC samples were from 26 randomly selected cancer patients. One specimen of SCC came from case 2 for whom a specimen of mild dysplasia has already been selected in the group of premalignant lesions (metachronous presentation).

### Telomerase assay

Frozen tissue samples, as small as 2 mm in diameter and for which a histological control was performed, were homogenized in 100 µl of lysis buffer (CHAPS) and incubated for 30 min at 4˚C. The lysates were centrifuged at 16 000 ***g*** for 20 min at 4˚C and supernatants were transferred into fresh Eppendorf tubes and stored at –80˚C until use. Protein concentration was carefully measured twice in each extract using the Bio-Rad Protein Assay (Bio-Rad Laboratories, California). Telomerase activities were assessed using the TRAPeze ELISA Telomerase detection kit (Oncor, Gaitherburg, MD) according to manufacturer's instructions. Assays have been performed twice in independent experiments using for each sample a constant amount of 0.1 µg of lysates.

In brief, when using the TRAPeze ELISA Telomerase detection kit (Oncor), lysates were incubated for the first steps in presence of a biotinylated telomerase substrate oligonucleotide (b-TS) at 30˚C for 30 min. Then, the extended products were amplified by a polymerase chain reaction (PCR) using Taq polymerase (Pharmacia Biotech, Upsala, Sweden), the b-TS, RP primers and a deoxynucleotide mix containing dCTP labelled with dinitrophenyl (DNP). The PCR conditions were 33 cycles of 94˚C for 30 s and 55˚C for 30 s. After PCR, the TRAP products were tagged with biotin and DP residues. The labelled products were immobilized onto streptavidin-coated microtitre plates via biotin-streptavidin interaction, and then detected by anti-DNP antibody conjugated to horseradish peroxidase (HPR). The amount of TRAP product was evaluated after addition of the peroxidase substrate (3,3′,5,5′-tetramethylbenzidine or TMB) by measuring absorbance at 450 nm and 595 nm (Microplate Reader 3550, Bio-Rad Laboratories). Our ELISA results were confirmed by a direct visualization of the TRAP ladder by a 12.5% non denaturing polyacrylamide gel electrophoresis.

Assays have been performed twice in independent experiments using a constant amount of 0.1 µg of lysates, for each sample that permits obtaining DO values in the linear range of the assay (Haik et al, 2000). Each sample was tested along with a heat inactivated (65˚C for 10 min) or RNase-treated aliquot. Reagent controls which lacked cell extracts were also systematically performed. According to the manufacturer's instruction absorbance of the inactivated sample was to be less than 0.25 in order to consider any result as accurate, and a sample was considered to be positive if the increase of absorbance: Ai (i.e. Absorbance of sample – Absorbance of inactivated sample) was over 0.15. The positive tumours (Ai > 0.15) were then ranked in two categories depending on the increase of absorbance. When Ai was between 0.15 and 0.5, the sample was considered to express a low telomerase level, and when Ai was over 0.5 the sample was defined as having a high telomerase level.

### Immunohistochemistry: detection of p16, cyclin D1, pRb and Ki67

Immunohistochemical stains were performed using a mouse monoclonal anti-p16 clone DCS-50.1/A7 (Neomarker, Union City, CA), a mouse monoclonal anti-cyclin D1 clone DCS-6 (Neomarker, Union City, CA), a rabbit polyclonal anti-Ki 67 (Dako, Trappes, France) and a mouse monoclonal anti-pRb clone G3-245 (Pharmingen, Los Angeles, CA). Deparaffinized sections were microwaved at 600 Watts 3 times for 5 min in 250 ml of 0.01 M sodium citrate buffer pH 6.0, and allowed to cool at room temperature for 20 min. The following steps were performed in an automated immunohistochemical processor (Ventana, Tucson, Arizona). Primary antibodies were incubated at 37˚C for 30 min (p16, pRb, Ki67: dilution 1:200; cyclin D1: dilution 1:150). For detection of all 4 bound antibodies, we used the Enhanced DAB Detection kit (Ventana, Tucson, Arizona). Slides were finally counterstained with Harris haematoxylin, and mounted. Normal epithelial tissue samples from the tonsil were used as an external positive control for the 4 antibodies, and were also stained with omission of the primary antibody in order to confirm staining-specificity. In addition the F9605 cell line was used as a negative external control for p16 (Gruis et al, 1995). Non-neoplastic stromal cells served as internal positive controls for pRb and p16 in each biopsy sample.

Specimens were blindly evaluated by a pathologist (PF) and another investigator (J-CS). Nuclear staining was used to identify positive cells. Representative areas of each tissue section were selected, and cells were counted in at least 4 fields (at × 400) in these areas. In each field at least 200 tumour cells were evaluated for each stain. Intensity of positive staining was graded as minimal, moderate, and strong. Absolute percentage of positive cells were recorded. Cases with a difference of more than 10% between observers were reviewed. Finally, average percentage was taken for each stain.

© 2001 Cancer Research Campaign 

### HPV detection

HPV detection was performed by means of PCR using E6-directed consensus primers and 32P-kinased type-specific oligonucleotide probes for types 16, 18, 31 and 33 as described elsewhere (Resnick et al, 1990). For each sample, 4 sections of 10 μm were used for HPV detection. Negative control samples and positive control samples from the uterine cervix known to contain various types were included in each amplification. PCR products from positive samples were also used to provide a control for each hybridization.

### Statistical analysis

Cases were classified as positive for telomerase activity if they demonstrated telomerase activity levels above the 0.15 level, as stated by the manufacturer. A complete absence of p16 or pRb staining was required to identify negative cases. Overexpression of cyclin D1 or Ki67 was determined using the median of the percentage of positive cells as a cut-off.

To evaluate trends in the percentage of positive cases for each variable as defined above with regard to severity of disease, lesion types were ordered according to their severity as mild dysplasia, advanced (moderate or severe) dysplasia and carcinoma, and assigned a score of 0, 1, and 2 respectively. Trends in proportions were identified using the Mann-Whitney U test, which is based on ranks and is applicable to very small samples (Moses et al, 1984).

All analyses were carried out with StatView software version 4.02. A level of 0.05 was chosen to indicate statistical significance. All reported *P* values are two-sided.

### RESULTS

### Telomerase activity

Telomerase-positive samples showed the characteristic processive 6-bp ladder upon PAGE (Figure 1). RNAse treatment or heat inactivation of cell extracts completely eliminated the signal demonstrating the specificity of the enzymatic detection.

The results of the TRAPeze ELISA Telomerase detection assay are shown in Table 1. Of a total of 48 samples 43 (90%) exhibited telomerase activity. High (>0.5) levels of telomerase activity were observed in 37 samples (77%).

### Immunohistochemistry of p16, cyclin D1, pRb and Ki67 proteins

The results of immunohistochemical analysis of p16, pRb, Ki67 and cyclin D1 expression are summarized in Table 1. Figure 2 shows examples of immunohistochemical staining for p16 (A and B), cyclin D1 (C and D), pRb (E) and Ki67 (F). Strong nuclear staining for p16 was sometimes associated with a weak to moderate cytoplasmic staining. Specificity of the immunoreactivity was confirmed by negative and internal or external positive controls for each antibody (see mesenchymal cells positive for p16 in Figure 2A and B) as described in methods.

34 of 48 (71%) lesions were totally unreactive with the p16 antibody. Lesions with p16 expression showed considerable heterogeneity in the proportion of positive cells (2 to 99%). All cases regarded as p16-negative had at least a few positive stromal cells as an internal positive control, therefore, validating p16 staining.

In contrast to p16 which was frequently negative, all lesions but one (98%) showed pRb immunoreactivity. The pRb staining was strong in all positive samples, although the proportion of positive cells extended from 3 to 95%.

All samples were strongly positive for Ki67. The median of the percentage of positive cells was 41.25% (range 13.5 to 80%).

43 (90%) of 48 samples were immunoreactive with the cyclin D1 antibody. The median of the percentage of positive cells was 27% (range 0–66.5%). There was some heterogeneity of intensity of staining, which was strong in 17 cases (35%), moderate in 14 cases (29%), and weak in 12 cases (35%).

### HPV status

This study identified HPV in 4 of 48 tested samples (8.3%) (Table 1). Among these HPV-positive samples, there was one mild dysplasia, one advanced dysplasia and two SCC. Three cases were positive for the high-risk HPV type 16, while the remaining case was positive for HPV type 31. Interestingly, among premalignant lesions, the sample (case 3, mild dysplasia) demonstrating the highest telomerase activity was HPV-positive for type 16, and the sample (case 14, advanced dysplasia) exhibiting the highest percentage of p16 positive cells (97.5%) was also HPV-positive.

### Telomerase activity, p16, cyclin D1 and Ki67 status according to severity of lesions

Proportions of cases demonstrating high levels of telomerase activity, expressing p16 or overexpressing cyclin D1 or Ki67 according to lesion types are shown in Figure 3.

Of 9 mild dysplasia, 5 (55%) demonstrated telomerase activity, and in 4 (44%) levels of telomerase activity were high (>0.5). 12 of 12 (100%) advanced dysplasia and 26 of 26 (100%) SCC exhibited telomerase activity, and the activity levels were high in 8 of

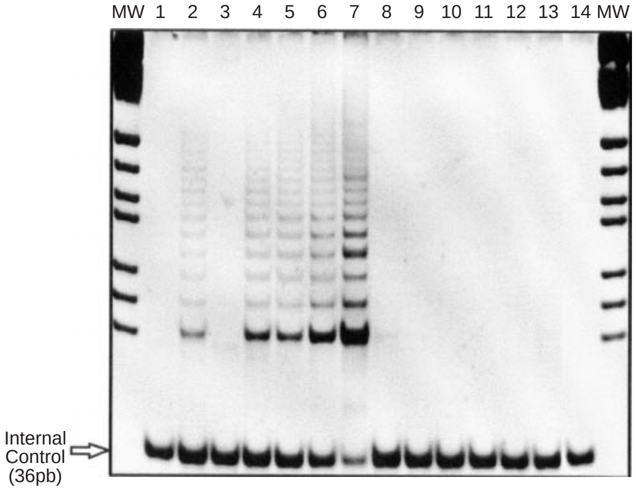

**Figure 1** Detection of telomerase activity in cell extracts from premalignant and malignant lesions. Polyacrylamide gel electrophoresis separation of telomerase ladder. Lanes 1 and 2: patient no. 2 (light dysplasia and squamous-cell carcinoma); lanes 3 and 4: patient no. 8 (light dysplasia and moderate dysplasia); lane 5: patient no. 24; lane 6: patient no. 43; lane 7: positive control; lane 8: lysis buffer; lanes 9 to 14: respective RNase-inactivated controls; lanes MW: molecular weight marker

© 2001 Cancer Research Campaign

**Table 1** Summary of patient characteristics, lesion type, immunohistochemistry of p16, cyclin D1, Ki 67, and pRb proteins, HPV typing, and telomerase activity assay in 21 premalignant and 27 malignant lesions of the head and neck

| Patient no. | Age | Sex | Lesion type | Telomerase (intensity) | P16 | Cyclin D1 | Ki67 | Rb | HPV type |
|---|---|---|---|---|---|---|---|---|---|
| 1 | 59 | M | Mild dysplasia | High | Retained | Overexpressed | High | Retained | Negative |
| 2[a] | 63 | M | Mild dysplasia | Negative | Lost | Low | Low | Retained | Negative |
| 3 | 79 | F | Mild dysplasia | High | Retained | Overexpressed | Low | Retained | 16 |
| 4 | 67 | M | Mild dysplasia | High | Retained | Low | High | Retained | Negative |
| 5 | 58 | M | Mild dysplasia | Low | Retained | Low | High | Retained | Negative |
| 6 | 63 | M | Mild dysplasia | Negative | Retained | Low | Low | Retained | Negative |
| 7 | 55 | M | Mild dysplasia | Negative | Retained | Low | Low | Retained | Negative |
| 8[b] | 58 | M | Mild dysplasia | Negative | Lost | Low | Low | Retained | Negative |
| 9 | 52 | F | Mild dysplasia | High | Lost | Low | Low | Retained | Negative |
| 10 | 51 | M | Moderate dysplasia | Low | Lost | Low | Low | Retained | Negative |
| 11 | 50 | F | Moderate dysplasia | High | Retained | Low | Low | Retained | Negative |
| 12 | 57 | M | Moderate dysplasia | High | Lost | Low | High | Retained | Negative |
| 13 | 71 | M | Moderate dysplasia | Low | Lost | Low | Low | Retained | Negative |
| 14 | 59 | M | Moderate dysplasia | High | Retained | Low | High | Retained | 16 |
| 15 | 61 | M | Moderate dysplasia | Low | Lost | Low | Low | Retained | Negative |
| 8[a] | 56 | M | Moderate dysplasia | High | Lost | Low | Low | Retained | Negative |
| 16 | 47 | M | Severe dysplasia | High | Retained | Overexpressed | Low | Retained | Negative |
| 17 | 84 | M | Severe dysplasia | High | Lost | Overexpressed | Low | Retained | Negative |
| 18 | 42 | M | Severe dysplasia | High | Lost | Overexpressed | High | Retained | Negative |
| 19 | 50 | M | Severe dysplasia | High | Lost | Low | Low | Retained | Negative |
| 20 | 46 | M | Severe dysplasia | Low | Retained | Low | High | Retained | Negative |
| 21 | 37 | M | Carcinoma | High | Lost | Low | High | Retained | Negative |
| 22 | 41 | F | Carcinoma | High | Retained | Low | Low | Retained | Negative |
| 23 | 72 | F | Carcinoma | High | Retained | Low | High | Retained | Negative |
| 24 | 72 | M | Carcinoma | High | Lost | Overexpressed | Low | Retained | 16 |
| 25 | 46 | M | Carcinoma | High | Lost | Overexpressed | High | Retained | Negative |
| 26 | 49 | M | Carcinoma | High | Lost | Overexpressed | High | Retained | Negative |
| 27 | 57 | M | Carcinoma | High | Lost | Low | Low | Retained | Negative |
| 28 | 45 | M | Carcinoma | Low | Lost | Overexpressed | Low | Retained | Negative |
| 29 | 45 | M | Carcinoma | High | Lost | Low | Low | Retained | Negative |
| 30 | 60 | M | Carcinoma | High | Lost | Overexpressed | High | Retained | Negative |
| 31 | 63 | F | Carcinoma | High | Lost | Low | High | Retained | Negative |
| 32 | 62 | M | Carcinoma | Low | Lost | Overexpressed | High | Retained | Negative |
| 33 | 62 | F | Carcinoma | High | Lost | Overexpressed | High | Retained | Negative |
| 34 | 51 | M | Carcinoma | High | Lost | Low | Low | Retained | Negative |
| 35 | 44 | M | Carcinoma | High | Lost | Overexpressed | High | Retained | Negative |
| 2[b] | 63 | M | Carcinoma | High | Lost | Overexpressed | Low | Retained | Negative |
| 36 | 67 | M | Carcinoma | High | Lost | Low | High | Retained | Negative |
| 37 | 48 | M | Carcinoma | High | Lost | Overexpressed | High | Retained | Negative |
| 38 | 49 | F | Carcinoma | High | Lost | Overexpressed | High | Retained | Negative |
| 39 | 62 | M | Carcinoma | High | Lost | Overexpressed | High | Retained | Negative |
| 40 | 49 | M | Carcinoma | High | Retained | Overexpressed | Low | Retained | Negative |
| 41 | 68 | F | Carcinoma | High | Lost | Overexpressed | Low | Retained | Negative |
| 42 | 59 | M | Carcinoma | High | Retained | Overexpressed | High | Retained | Negative |
| 43 | 75 | M | Carcinoma | High | Lost | Overexpressed | Low | Retained | Negative |
| 44 | 58 | M | Carcinoma | High | Lost | Overexpressed | High | Lost | Negative |
| 45 | 54 | M | Carcinoma | High | Lost | Low | High | Retained | 31 |
| 46 | 70 | F | Carcinoma | High | Lost | Overexpressed | High | Retained | Negative |

Patients 2 and 8 are shown twice as they presented consecutively with different lesions (metachronous presentation).

12 (67%) advanced dysplasia and all but two (92%) SCC. The trend of increasing proportions of cases demonstrating high levels of telomerase activity with severity of lesions was significant ($P$ = 0.009).

Mild or moderate staining for cyclin D1 in suprabasal cells was characteristic of mild dysplasia (Figure 2C), while strong staining was observed in a majority of tumour cells in SCC (Figure 2D). The trend of increasing proportions of cases overexpressing cyclin D1 (above the median) with severity of lesions was significant ($P$ = 0.02) as 2 of 9 mild dysplasia (22%), 4 of 12 (33%) advanced dysplasia, and 18 of 27 (66%) SCC samples overexpressed cyclin D1. Analysis of cyclin D1 staining intensity also showed that strong staining was observed in none of 9 (0%) mild dysplasia, in 4 of 12 (25%) advanced dysplasia, and 13 of 27 (48%) SCC ($P$ = 0.01).

In contrast to staining with cyclin D1, most SCC were p16 negative (Figure 2B). An inverse trend of decreasing proportions of cases expressing p16 with severity of lesions was observed ($P$ = 0.01). 6 of 9 (67%) mild dysplasia displayed p16 expression with a wide range from 3.5 to 59% positive epithelial cells, as well as 4 of 12 (33%) advanced dysplasia (Figure 2A). Only 4 of 27 (15%) SCC were p16-positive, although the proportion of positive tumour cells could reach 99% in these cases.

Overexpression of Ki67 was found in 3 of 9 mild dysplasia (33%), 4 of 12 advanced dysplasia (33%) and 17 of 27 SCC (63%; Figure 2F), but this trend was not significant ($P$ = 0.08).

© 2001 Cancer Research Campaign 

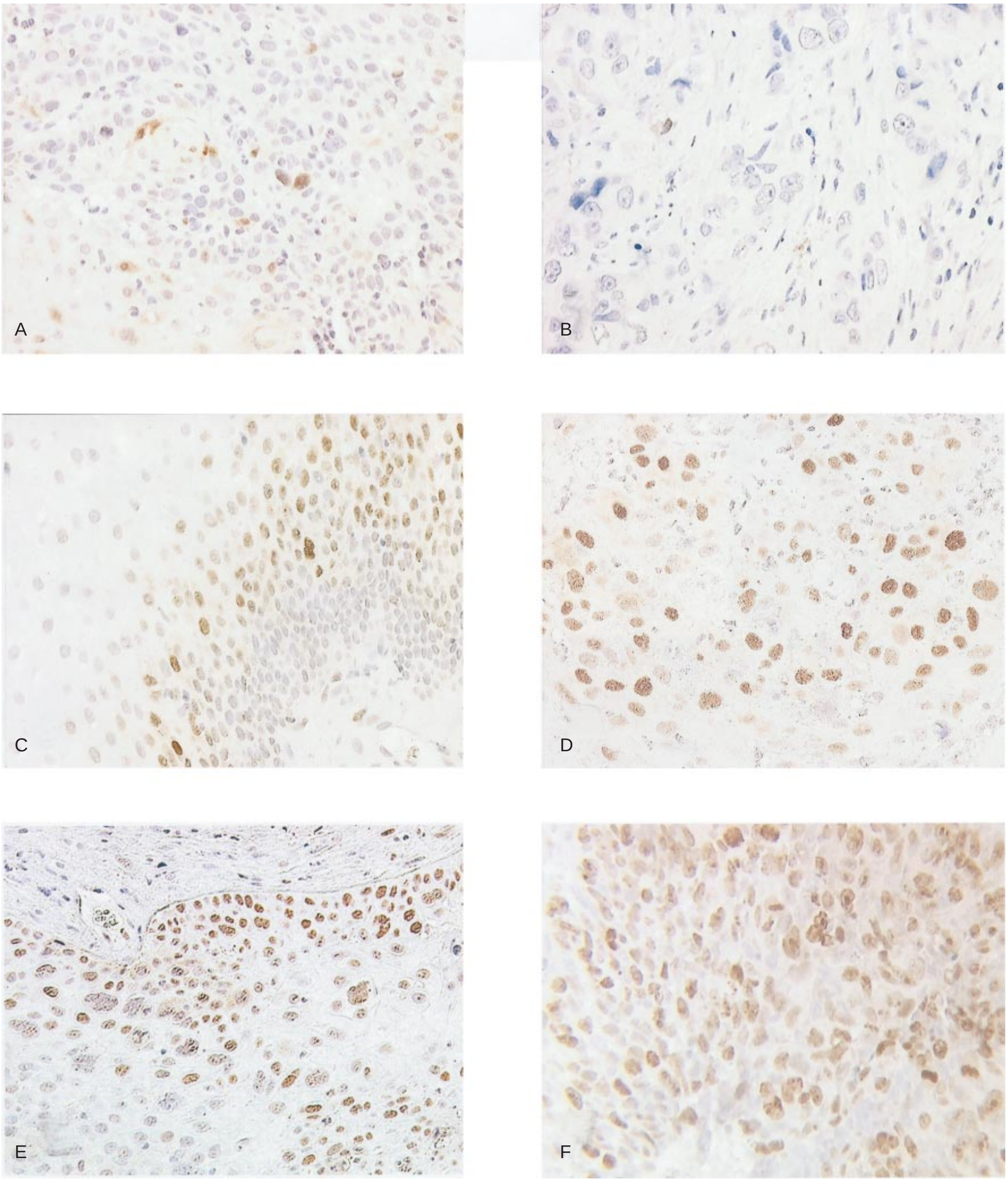

**Figure 2** Examples of immunoreactivity with the p16 (**A–B**), cyclin D1 (**C–D**), pRb (**E**) and Ki67 (**F**) antibodies. (**A**) Focal positivity for p16 in a case of advanced dysplasia (patient no. 16), (**B**) complete lack of p16 reactivity in tumour cells in a case of squamous-cell carcinoma (patient no. 26), (**C**) suprabasal expression of cyclin D1 in a case of mild dysplasia/keratosis (patient no. 4), (**D**) strong and diffuse staining for cyclin D1 in a case of squamous-cell carcinoma (patient no. 32), (**E**) a dysplasia showing a strong positive reaction in the nuclei of cells for pRb (patient no. 18), (**F**) a squamous-cell carcinoma with most carcinoma cells positive for Ki-67 (patient no. 45)

### DISCUSSION

A majority of SCCHN are known to demonstrate high telomerase activity, inactivation of p16, and/or overexpression of cyclin D1 (Reed et al, 1996; Jares et al, 1997; Olshan et al, 1997). Moreover, high telomerase activity (Mao et al, 1996; Mutirangura et al, 1996), or inactivation of p16 (Papadimitrakopoulou et al, 1997; Pande et al, 1998), or overexpression of cyclin D1 (Izzo et al, 1998) have been observed separately in a proportion of premalignant lesions of the head and neck epithelium demonstrating that these abnormalities may occur at the preinvasive stage of disease. Our results are clearly consistent with these observations. Our study is the first one to show in the same set of samples of premalignant and malignant lesions the parallel occurrence of the most frequently encountered oncogenic changes involving cell cycle regulators (p16, cyclin D1) together with telomerase

© 2001 Cancer Research Campaign

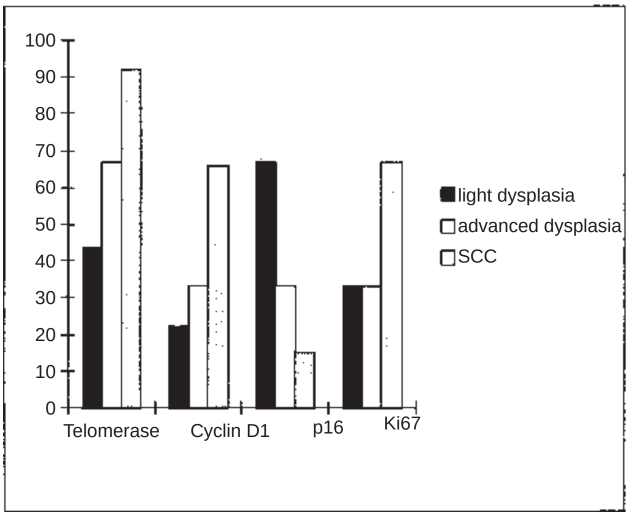

**Figure 3** Proportions of cases demonstrating high levels of telomerase activity, expressing p16 or overexpressing cyclin D1 or Ki67 according to lesion types. The trends in proportions were found significant for telomerase activity ($P$ = 0.009), overexpression of cyclin D1 ($P$ = 0.02) and expression p16 ($P$ = 0.01)

activation. Telomerase activation and p16 disruption are both required for the immortalisation process of epithelial cells in vitro models (Kiyono et al, 1998). Here in an in vivo study, we demonstrate that the tumorigenic progression in the head and neck epithelium is clearly associated with telomerase activation along with p16 inactivation.

Our findings were established in cases carefully selected to display identical histological presentation in contiguous frozen and paraffin-embedded parts. The antibodies for pRb and p16 were chosen among other commercially available reagents because there were previous studies in the literature showing a good concordance between immunoreactivity with these reagents in tissue sections and the results of parallel genetic analysis (Geradts et al, 1994; Bartkova et al, 1995; Reed et al, 1996). These studies have concluded to the value of using immunohistochemistry for assessing small lesions for which sufficient DNA is not available for detailed genetic analysis. Moreover, the F9605 cell line which is homozygously deleted at the INK4a locus (Gruis et al, 1995) was assayed as an external negative control for the p16 antibody. An advantage of the DCS 50.1 antibody over other antibodies for p16 is that sample negativity is unambiguously defined by the complete absence of positive cells. Epitope masking or lability can be important problems in paraffin-embedded sections, however. The frequency of p16-negative SCC in our series (85%) was very close to the previously reported frequency (83%) in the series of Reed et al, which was performed on frozen specimens and showed a good concordance between immunoreactivity and genetic lesions. We avoided overfixation by limiting the length of time in fixative to less than 24 hours for all cases. Sections were made 2–3 weeks prior to performing the immunostaining. Importantly, there was a positive internal control for pRb or p16 in each case, which clearly demonstrates that these epitopes were detectable in tissue sections even though tumour cells were negative. Wu et al also found a high frequency, (87%) of p16 loss in a series of oral and oropharyngeal carcinomas and concluded that DNA studies may underestimate the true prevalence of p16 loss (Wu et al, 1999). Overexpression of cyclin D1 in specimens analysed by immunohistochemistry also indicates that DNA analysis may underestimate the frequency of cyclin D1 abnormalities (Bartkova et al, 1995). On the other hand, missense mutations of p16 may well stain using immunohistochemistry (Zhang et al, 1994; Munro et al, 1999), although the rate of point mutations of p16 is generally low in SCCHN (Zhang et al, 1994; Reed et al, 1996; Gazzeri et al, 1998; Sanchez-Cespedes et al, 1999). Tumour 44, which had no p16 and no pRb, is an intriguing case. Immunostaining for p16 or pRb was clearly positive with nuclear staining on many stromal cells. Tumour cells were completely unstained by pRb antibody. However, they contained a moderate to strong amount of cytoplasmic p16 without concomitant nuclear staining. This case was thus considered to be p16-negative. It is true that p16 and pRb mutations are almost always mutually exclusive (Kinoshita et al, 1996; Kratzke et al, 1996). However, a few tumours have been described with p16 and pRb alterations suggesting that either one of these genetic changes may lead to an additional growth advantage for the transformed cell (Sanchez-Cespedes et al, 1999). The INK4a locus harbours both p16 and p14/ARF genes (Quelle et al, 1995). Specific inactivation of the p16/pRb pathway relates to evasion of senescence, while p14/ARF regulates the p53 pathway (Munro et al, 1999). It is conceivable that the pRb pathway may be disabled through loss of pRb, while alteration at the INK4a locus represents an additional oncogenic event targeting the p53 pathway. Exclusive cytoplasmic p16 staining has been related to a more aggressive behaviour in breast cancer (Emig et al, 1998). Case 44 was a poorly differentiated SCCHN with a high mitotic count and a high percentage of Ki67 positive cells. However, we could not document the follow-up in this case.

We analysed the telomerase activity of our samples using the TRAP method (Shay and Bacchetti, 1997). Unfortunately this technique does not allow an in situ analysis of telomerase activity, and the precise contribution of infiltrating lymphocytes is hard to ascertain regarding positive results. To our knowledge, there is no method fully reliable to directly evaluate telomerase activity at the cellular level. We have tried, as others, some commercially available antibodies against TERT, but without satisfactory results (Dhaene et al, 2000). In situ hybridization of TERT – the catalytic subunit of telomerase which is assumed to be directly responsible for activation of telomerase – might be an alternative while waiting for suitable anti-hTERT antibodies (Kolquist et al, 1998). However, even with this technique some authors were unable to find a strict correlation between telomerase activity and hTERT mRNA expression (Nakano et al, 1998). For these reasons, we analysed the telomerase activity of our samples using the TRAP method. Although the telomerase activity levels cannot be considered as quantitative due to the exponential nature of the PCR amplification, we and others (Sumida et al, 1999; Haik et al, 2000) have consistently shown that differences in telomerase activity levels can be shown reproducibly using the TRAP assay. Therefore we felt entitled to split telomerase-positive lesions in two groups of high and low level of expression depending on the increase of absorbance. The cut-off point of 0.5 in the increase of absorbance (Absorbance of sample – Absorbance of inactivated sample) is somewhat arbitrary, but it was the one reflecting the best visual evaluation of the TRAP-ELISA result (weak or strong colour reaction). As previously stated, since the TRAP assay is performed on a homogenate it is difficult to interpret our findings at the cellular level. However our results, when analysed in relation to histological changes, clearly show that the proportions of cases demonstrating high telomerase activity increase in a continuous manner with phenotypic progression from the earliest pathologically detectable premalignant lesions (keratose/mild

© 2001 Cancer Research Campaign dysplasia) to advanced premalignant lesions (moderate or severe dysplasia) and to subsequent invasive carcinoma (Figure 3). An increased telomerase activity with increasing head and neck cancer stage has been reported before (Mao et al, 1996; Mutirangura et al, 1996), and is reminiscent of TERT expression in human breast and colon tissues where it begins with early pre-invasive changes and increases gradually during tumour progression (Kolquist et al, 1998). This suggests a continuous selection of cells with higher telomerase activity during tumour progression in contrast to the late and stochastic activation by cells in culture. Of special interest are the two cases with metachronous presentation (cases 2 and 8). Indeed a transition from TRAP-negative to -positive results is observed in the biopsies. However the precise meaning of this observation remains elusive since it could either represent different stages in the development of the same tumour cell population or could be simply related to independent lesions.

The coincident loss of p16 and telomerase activation during tumour progression strongly suggests these events cooperate in conferring immortality to tumour cells. Telomerase activation and p16 disruption are required to immortalize epithelial cells (Kiyono et al, 1998). Keratinocyte immortalization can be achieved without affecting other major growth control or differentiation systems (Dickson et al, 2000), which is perfectly consistent in our study with the observation of telomerase activation and p16 loss in a low percentage of early lesions (keratoses/light dysplasia), which are defined by minimal disturbance of tissular differentiation and slight degree of atypia. However, cultures from different stages of SCCHN have revealed that immortality is a frequent, but dispensable event at a late stage during tumour progression (Edington et al, 1995). In addition to p16 loss and telomerase activation two pathways are involved to immortalize human SCC keratinocytes, which may explain why more carcinomas appear to be p16−/− and telomerase-positive than there are immortal SCCHN (Loughran et al, 1997). To prove that the coincident loss of p16 and telomerase activation in our cases do actually result in immortalization of tumour cells as expected would have required to relate this molecular profile to the appearance of immortal variants in cultures.

HPV infection affected less than 10% of our cases. The interference of HPV on telomerase activity or the pRb pathway is well demonstrated by experimental studies (Jones and Munger, 1996; Klingelhutz et al, 1996). We do not provide evidence that HPV is functionally significant since we do not demonstrate that there is at least one copy of the virus per cell, but our observations are consistent with experimental data.

In conclusion, our study shows that telomerase activation and the most commonly observed oncogenic changes involving cell cycle regulators accumulate continuously with disease progression in the head and neck tumorigenic process. A better evaluation of the relationships between these variables at the cellular level might provide additional information regarding the chronological organization of these events.

### ACKNOWLEDGEMENTS

We thank Dr Li Mao for his critical review of the manuscript and helpful advice. We are grateful to NA Gruis and N Smit for providing the F9605 cell line and G Le Naour for the artwork. This work was supported by the following grants: Contrat n°FIGH-CT 1999 (SUSGENESINRADCAR) from the European Community, contrat n° 98-RSRC21 from the Comité de Paris de la Ligue contre le Cancer; Fondation de France, AP-HP and Lilly Fondation Grant to J-CS.

### REFERENCES

Bartkova J, Lukas J, Muller H, Strauss M, Gusterson B and Bartek J (1995) Abnormal patterns of D-type cyclin expression and G1 regulation in human head and neck cancer. *Cancer Res* **55**: 949–956

Crissman JD and Zarbo RJ (1989) Dysplasia, in situ carcinoma, and progression to invasive squamous cell carcinoma of the upper aerodigestive tract. *Am J Surg Pathol* **13**: 5–16

Dellambra E, Golisano O, Bondanza S, Siviero E, Lacal P, Molinari M, D'Atri S and De Luca M (2000) Downregulation of 14–3–3sigma prevents clonal evolution and leads to immortalization of primary human keratinocytes. *J Cell Biol* **149**: 1117–1130

Dhaene K, Wauters J, Weyn B, Timmermans JP and van Marck E (2000) Expression profile of telomerase subunits in human pleural mesothelioma. *J Pathol* **190**: 80–85

Dickson MA, Hahn WC, Ino Y, Ronfard V, Wu JY, Weinberg RA, Louis DN, Li FP and Rheinwald JG (2000) Human keratinocytes that express hTERT and also bypass a p16(INK4a)-enforced mechanism that limits life span become immortal yet retain normal growth and differentiation characteristics. *Mol Cell Biol* **20**: 1436–1447

Edington KG, Loughran OP, Berry IJ and Parkinson EK (1995) Cellular immortality: a late event in the progression of human squamous cell carcinoma of the head and neck associated with p53 alteration and a high frequency of allele loss. *Mol Carcinog* **13**: 254–265

Emig R, Magener A, Ehemann V, Meyer A, Stilgenbauer F, Volkmann M, Wallwiener D and Sinn HP (1998) Aberrant cytoplasmic expression of the p16 protein in breast cancer is associated with accelerated tumour proliferation. *Br J Cancer* **78**: 1661–1668

Gazzeri S, Gouyer V, Vour'ch C, Brambilla C and Brambilla E (1998) Mechanisms of p16INK4A inactivation in non small-cell lung cancers. *Oncogene* **16**: 497–504

Geradts J, Hu SX, Lincoln CE, Benedict WF and Xu HJ (1994) Aberrant RB gene expression in routinely processed, archival tumor tissues determined by three different anti-RB antibodies. *Int J Cancer* **58**: 161–167

Greenberg RA, Chin L, Femino A, Lee KH, Gottlieb GJ, Singer RH, Greider CW and DePinho RA (1999) Short dysfunctional telomeres impair tumorigenesis in the INK4a(delta2/3) cancer-prone mouse. *Cell* **97**: 515–525

Gruis NA, van der Velden PA, Sandkuijl LA, Prins DE, Weaver-Feldhaus J, Kamb A, Bergman W and Frants RR (1995) Homozygotes for CDKN2 (p16) germline mutation in Dutch familial melanoma kindreds. *Nat Genet* **10**: 351–353

Haik S, Gauthier LR, Granotier C, Peyrin JM, Lages CS, Dormont D and Boussin FD (2000) Fibroblast growth factor 2 upregulates telomerase activity in neural precursor cells. *Oncogene* **19**: 2957–2966

Huschtscha LI and Reddel RR (1999) p16(INK4a) and the control of cellular proliferative life span. *Carcinogenesis* **20**: 921–926

Izzo JG, Papadimitrakopoulou VA, Li XQ, Ibarguen H, Lee JS, Ro JY, El-Naggar A, Hong WK and Hittelman WN (1998) Dysregulated cyclin D1 expression early in head and neck tumorigenesis: in vivo evidence for an association with subsequent gene amplification. *Oncogene* **17**: 2313–2322

Jares P, Fernandez PL, Nadal A, Cazorla M, Hernandez L, Pinyol M, Hernandez S, Traserra J, Cardesa A and Campo E (1997) p16MTS1/CDK4I mutations and concomitant loss of heterozygosity at 9p21–23 are frequent events in squamous cell carcinoma of the larynx. *Oncogene* **15**: 1445–1453

Jones DL and Munger K (1996) Interactions of the human papillomavirus E7 protein with cell cycle regulators. *Semin Cancer Biol* **7**: 327–337

Kinoshita I, Dosaka-Akita H, Mishina T, Akie K, Nishi M, Hiroumi H, Hommura F and Kawakami Y (1996) Altered p16INK4 and retinoblastoma protein status in non-small cell lung cancer: potential synergistic effect with altered p53 protein on proliferative activity. *Cancer Res* **56**: 5557–5562

Kiyono T, Foster SA, Koop JI, McDougall JK, Galloway DA and Klingelhutz AJ (1998) Both Rb/p16INK4a inactivation and telomerase activity are required to immortalize human epithelial cells [see comments]. *Nature* **396**: 84–88

Klingelhutz AJ, Foster SA and McDougall JK (1996) Telomerase activation by the E6 gene product of human papillomavirus type 16. *Nature* **380**: 79–82

Kolquist KA, Ellisen LW, Counter CM, Meyerson M, Tan LK, Weinberg RA, Haber DA and Gerald WL (1998) Expression of TERT in early premalignant lesions and a subset of cells in normal tissues. *Nat Genet* **19**: 182–186

 © 2001 Cancer Research Campaign

Kratzke RA, Greatens TM, Rubins JB, Maddaus MA, Niewoehner DE, Niehans GA and Geradts J (1996) Rb and p16INK4a expression in resected non-small cell lung tumors. *Cancer Res* **56**: 3415–3420

Lin AW, Barradas M, Stone JC, van Aelst L, Serrano M and Lowe SW (1998) Premature senescence involving p53 and p16 is activated in response to constitutive MEK/MAPK mitogenic signaling. *Genes Dev* **12**: 3008–3019

Loughran O, Clark LJ, Bond J, Baker A, Berry IJ, Edington KG, Ly IS, Simmons R, Haw R, Black DM, Newbold RF and Parkinson EK (1997) Evidence for the inactivation of multiple replicative lifespan genes in immortal human squamous cell carcinoma keratinocytes. *Oncogene* **14**: 1955–1964

Mao L, El-Naggar AK, Fan YH, Lee JS, Lippman SM, Kayser S, Lotan R and Hong WK (1996) Telomerase activity in head and neck squamous cell carcinoma and adjacent tissues. *Cancer Res* **56**: 5600–5604

Moses LE, Emerson JD and Hosseini H (1984) Analyzing data from ordered categories. *N Engl J Med* **311**: 442–448

Munro J, Stott FJ, Vousden KH, Peters G and Parkinson EK (1999) Role of the alternative INK4A proteins in human keratinocyte senescence: evidence for the specific inactivation of p16INK4A upon immortalization. *Cancer Res* **59**: 2516–2521

Mutirangura A, Supiyaphun P, Trirekapan S, Sriuranpong V, Sakuntabhai A, Yenrudi S and Voravud N (1996) Telomerase activity in oral leukoplakia and head and neck squamous cell carcinoma. *Cancer Res* **56**: 3530–3533

Nakano K, Watney E and McDougall JK (1998) Telomerase activity and expression of telomerase RNA component and telomerase catalytic subunit gene in cervical cancer. *Am J Pathol* **153**: 857–864

Okami K, Reed AL, Cairns P, Koch WM, Westra WH, Wehage S, Jen J and Sidransky D (1999) Cyclin D1 amplification is independent of p16 inactivation in head and neck squamous cell carcinoma. *Oncogene* **18**: 3541–3545

Olshan AF, Weissler MC, Pei H, Conway K, Anderson S, Fried DB and Yarbrough WG (1997) Alterations of the p16 gene in head and neck cancer: frequency and association with p53, PRAD-1 and HPV. *Oncogene* **14**: 811–818

Pande P, Mathur M, Shukla NK and Ralhan R (1998) pRb and p16 protein alterations in human oral tumorigenesis. *Oral Oncol* **34**: 396–403

Papadimitrakopoulou V, Izzo J, Lippman SM, Lee JS, Fan YH, Clayman G, Ro JY, Hittelman WN, Lotan R, Hong WK and Mao L (1997) Frequent inactivation of p16INK4a in oral premalignant lesions. *Oncogene* **14**: 1799–1803

Planas-Silva MD and Weinberg RA (1997) The restriction point and control of cell proliferation. *Curr Opin Cell Biol* **9**: 768–772

Quelle DE, Zindy F, Ashmun RA and Sherr CJ (1995) Alternative reading frames of the INK4a tumor suppressor gene encode two unrelated proteins capable of inducing cell cycle arrest. *Cell* **83**: 993–1000

Reed AL, Califano J, Cairns P, Westra WH, Jones RM, Koch W, Ahrendt S, Eby Y, Sewell D, Nawroz H, Bartek J and Sidransky D (1996) High frequency of p16 (CDKN2/MTS-1/INK4A) inactivation in head and neck squamous cell carcinoma. *Cancer Res* **56**: 3630–3633

Resnick RM, Cornelissen MT, Wright DK, Eichinger GH, Fox HS, ter Schegget J and Manos MM (1990) Detection and typing of human papillomavirus in archival cervical cancer specimens by DNA amplification with consensus primers. *J Natl Cancer Inst* **82**: 1477–1484

Sanchez-Cespedes M, Reed AL, Buta M, Wu L, Westra WH, Herman JG, Yang SC, Jen J and Sidransky D (1999) Inactivation of the INK4A/ARF locus frequently coexists with TP53 mutations in non- small cell lung cancer. *Oncogene* **18**: 5843–5849

Serrano M, Lee H, Chin L, Cordon-Cardo C, Beach D and DePinho RA (1996) Role of the INK4a locus in tumor suppression and cell mortality. *Cell* **85**: 27–37

Serrano M, Lin AW, McCurrach ME, Beach D and Lowe SW (1997) Oncogenic ras provokes premature cell senescence associated with accumulation of p53 and p16INK4a. *Cell* **88**: 593–602

Shay JW and Bacchetti S (1997) A survey of telomerase activity in human cancer. *Eur J Cancer* **33**: 787–791

Sumida T, Hamakawa H, Sogawa K, Sugita A, Tanioka H and Ueda N (1999) Telomerase components as a diagnostic tool in human oral lesions. *Int J Cancer* **80**: 1–4

Wu CL, Roz L, McKown S, Sloan P, Read AP, Holland S, Porter S, Scully C, Paterson I, Tavassoli M and Thakker N (1999) DNA studies underestimate the major role of CDKN2A inactivation in oral and oropharyngeal squamous cell carcinomas. *Genes Chromosomes Cancer* **25**: 16–25

Yoo GH, Xu HJ, Brennan JA, Westra W, Hruban RH, Koch W, Benedict WF and Sidransky D (1994) Infrequent inactivation of the retinoblastoma gene despite frequent loss of chromosome 13q in head and neck squamous cell carcinoma. *Cancer Res* **54**: 4603–4606

Zhang SY, Klein-Szanto AJ, Sauter ER, Shafarenko M, Mitsunaga S, Nobori T, Carson DA, Ridge JA and Goodrow TL (1994) Higher frequency of alterations in the p16/CDKN2 gene in squamous cell carcinoma cell lines than in primary tumors of the head and neck. *Cancer Res* **54**: 5050–5053

Zhu J, Woods D, McMahon M and Bishop JM (1998) Senescence of human fibroblasts induced by oncogenic Raf. *Genes Dev* **12**: 2997–3007

*© 2001 Cancer Research Campaign*
